# Subjective Monitoring in Under-20 Soccer Athletes: Affective, Load, Monotony, and Sleep Variations throughout a Competitive Cycle

**DOI:** 10.2174/0117450179415875251006053421

**Published:** 2025-10-09

**Authors:** Alessandro O. F. Junior, Ismael Viana Caldeira, Alberto Souza Sá Filho, Dailson P. Silva, Igor B. de Souza, Lorhenna P. Souza, Pedro Augusto Inacio, Gustavo de Conti Teixeira Costa, Vicente Aprigliano, Gaspar R. Chiappa, Sergio Machado, Marcelo Magalhães Sales, Eduardo M.M. Portugal

**Affiliations:** 1 Exercise Psychophysiology Laboratory - Laboratório de Psicofisiologia do Exercício (LaPE), Federal University of Rio de Janeiro (UFRJ), Rio de Janeiro, Brazil; 2 Biometry Laboratory - Laboratório de Biometria, Ladebio, Rio de Janeiro, Brazil; 3 Graduate Program at the Evangelical University of Goiás (UniEVANGÉLICA), Anápolis, Goiás, GO, Anápolis, Goiás, GO, Brazil; 4 Physiology Department of Al Nassr Football Club, Riyadh, Saudi Arabia; 5 Graduate Program at the Federal University of Goiás (UFG), Campus Samambaia, Goiânia, Goiás, Brazil; 6 Escuela de Ingeniería de Construcción y Transporte, Pontificia Universidad Catolica de Valparaíso, Avda Brasil 2147, Valparaíso 2362804, Chile; 7 Faculty of Health Sciences, Universidad Autónoma de Chile, Providencia, Santiago, 7500912, Chile; 8 Center of Neuroscience, Neurodiversity Institute, Queimados- RJ , Brazil; 9 LABPR – Panic and Respiration Laboratory (LABPR) - Institute of Psychiatry (IPUB), Federal University of Rio de Janeiro (UFRJ), Rio de Janeiro, Brazil; 10 Graduate Program in Environment and Society at the State University of Goiás – UEG, Southwest Campus, Quirinópolis, Goiás, Brazil

**Keywords:** Affect, Perceived exertion, Sleep, Monotony, Strain, Soccer, Youth athletes

## Abstract

**Introduction:**

The physical and mental demands of competitive soccer, combined with a high density of matches and training sessions, impose substantial psychophysiological stress on athletes. In this context, subjective variables, such as affective responses, perceived exertion, and sleep, emerge as important indicators, offering additional insights into players’ readiness and recovery. However, few studies have examined these variables in an integrated manner over extended periods of preparation and competition.

**Objective:**

This study aimed to investigate the effects of soccer training sessions and matches on affective responses (valence and arousal), perceived exertion, and sleep duration in under-20 soccer athletes. Additionally, the objective of this study was to assess correlations between affective responses and traditional internal load variables (RPE), monotony, strain, and self-reported sleep duration.

**Methods:**

This observational study was conducted with 21 under-20 athletes from a Brazilian elite soccer team over 11 weeks encompassing training sessions, friendly matches, and official competition. Affective responses were assessed before and 30 minutes after each session using the Feeling Scale (FS) and the Felt Arousal Scale (FAS). Perceived exertion (RPE), sleep duration, monotony, and strain were recorded daily. Two-way ANOVA was applied for FS and FAS, while one-way ANOVA was used for weekly training load, sleep, monotony, and strain. Mann-Whitney tests (match vs. training) were also performed. Pearson’s correlation coefficients were calculated between variables. The significance level was set at p < 0.05.

**Results:**

There were significant main effects of week and moment (pre/post) for both FS and FAS, with a notable decrease in affective responses after sessions (p < 0.0001). Sleep duration progressively increased from the seventh week onward (p < 0.05), whereas training load, monotony, and strain fluctuated across the weeks, with significant declines toward the end of the cycle. Significant differences between matches and training sessions were found for training load (p = 0.0333) and sleep duration (p < 0.0001), but not for affective scales. On an individual level, 71% of athletes showed a reduction in affective responses post-session. Correlations between affective and the other variables were trivial to small (ranging from r = 0.11 to r = 0.24), with slightly more consistent associations for sleep and RPE.

**Conclusion:**

There was a significant reduction in FS over the weeks. Fluctuations in FS were observed across weeks in line with accumulated load and competition demands. Seventy-one percent of athletes showed a decrease in FS post-activity. The monotony and strain showed a declining trend over the competitive cycle, particularly in the latter weeks, indicating a reduction in training variability and cumulative load. These reductions coincided with an increase in self-reported sleep duration, suggesting a favorable shift in the balance between training stress and recovery. Finally, FS displayed weak correlations with perceived exertion, monotony, strain, and sleep duration.

## 1 INTRODUCTION

Football seasons are characterized by high physical and psychological demands due to the accumulation of training sessions and competitive matches [1]. These demands impose significant psychophysiological strain on athletes, particularly during preparatory and congested periods of the season [1, 2]. Traditionally, monitoring strategies have focused on external load variables (*e.g.*, distance covered, sprint count) and internal load markers, such as heart rate and perceived exertion [3, 4]. Within internal load metrics, indicators, such as session rating of perceived exertion (RPE), monotony, and strain, have become essential for understanding the load-recovery balance and preventing overtraining [5, 6]. These strategies could help athletes to benefit not only from enhanced performance, but also from the maintenance or improvement of overall health. Recent evidence shows that regular physical activity at the right doses protects against mental health issues, such as depression and anxiety [7], and reduces the risk of severe outcomes in diseases [8]. These findings highlight the importance of monitoring not just for performance, but also for long-term psychophysiological responses.

Fessi *et al.* [9] monitored professional soccer players across pre-season and early season periods, reporting increased training loads to be associated with reduced sleep quality, elevated fatigue, and less positive affective states. Although their findings highlighted the interplay between psychophysical strain and wellness markers, the study relied on group-level trends, assessed valence only once per day, and did not explore within-subject variability. Moreover, their focus on adult athletes during predefined phases of the season limited the generalizability to youth athletes undergoing intense competitive cycles. These aspects underscore the need for studies that capture session-level affective dynamics, consider intraindividual variability, and integrate multiple subjective markers in real-world youth competition settings.

In this context, recent research suggests that affective responses may offer additional insight into how people experience and respond to exercise stimuli [10-14]. Tools, such as the Feeling Scale and Felt Arousal Scale, provide accessible measures of valence and activation, respectively, and can reflect the interplay among cognitive, interoceptive, and contextual factors in exercise environments [11, 15, 16]. Despite their established role in exercise science, affective responses have been underexplored in applied team sports settings, particularly within longitudinal monitoring of youth soccer athletes.

Studies on professional soccer have identified links among perceived exertion, training monotony, and wellness markers, such as sleep and mood [9, 17, 18]. Yet, the integration of affective responses into this multidimensional monitoring framework remains limited, and it is unclear how affective valence and activation fluctuate across weeks of training and competition, or how they relate to classic internal load variables. Moreover, although interindividual variability in affective responses is well documented [10, 11, 19], few studies have addressed this phenomenon within the context of high-stakes competitive cycles, such as the Copa São Paulo de Futebol Júnior (Brazil).

The aim of this study was to examine the impact of soccer training sessions and matches on pre- and post-affective responses, RPE, and sleep patterns in young soccer athletes. Secondarily, we analyzed the correlations between all variables collected in the present study. This investigation adds to the literature by integrating affective, perceptual, and recovery-related variables over an 11-week competitive period, with emphasis on intraindividual affective variability, an aspect still underexplored in youth soccer contexts. By capturing fluctuations in affect, perceived exertion, and sleep in response to training and match demands, the present study could offer ecologically relevant insights to inform individualized monitoring strategies. These findings may contribute to the early identification of maladaptive responses and to the adjustment of training loads in applied settings, particularly among young athletes exposed to congested schedules and elevated psychological demands during major competitions.

Based on these premises, the present study was designed to test the following hypotheses: (H1) training sessions and football matches influence affective responses, perceived effort and sleep patterns of young athletes; (H2) these responses are expected to fluctuate throughout the weeks, shaped by the cumulative training load and the demands of the competition; (H3) it is expected that affective responses are associated with perceived effort and sleep patterns, reflecting the interaction of psychophysiological and environmental factors.

## 2 MATERIALS AND METHODS

### 2.1 Experimental Approach

This study employed a longitudinal observational design with a quantitative approach and was conducted over an 11-week period involving under-20 male soccer athletes from a Brazilian elite team. This manuscript was prepared in accordance with the STROBE (Strengthening the Reporting of Observational Studies in Epidemiology) checklist [20], which guided the transparent and structured reporting of the study’s design. All participants were informed that the study involved routine monitoring with no impact on their training or recovery protocols. To avoid influencing behavior or coaching decisions, individual data were not disclosed during the study period. The study was approved by the research ethics committee of the Clementino Fraga Filho University Hospital, Brazil (protocol number: 4.661.552) and complied with the ethical principles outlined in the Declaration of Helsinki. All participants signed an informed consent form. For athletes under the age of 18, informed consent was also obtained from their legal guardians.

### 2.2 Sample

The present study was conducted with 21 male soccer athletes from the under-20 category of an elite Brazilian soccer over an 11-week period, which included training sessions, friendly matches, and the primary competition for their category in Brazil, called Copa São Paulo de Futebol Júnior. This competition holds critical importance as it serves as a platform for athletes to demonstrate their potential, influencing their future in professional soccer. While some players transition to the professional team after the competition, others remain in the under-20 category, and a few may be released from the club. Therefore, effective preparation and exceptional performance in this pivotal competition are crucial for these young athletes as they transition through this defining phase of their careers.

To be eligible for participation in the study, athletes were required to meet the following inclusion criteria: (1) be a registered member of the under-20 squad of the club during the entire observation period; (2) regularly participate in training sessions and competitive matches throughout the 11-week monitoring period; (3) be available for data collection at the designated times before and after sessions; and (4) provide informed consent, with additional consent from legal guardians in the case of participants under 18 years of age.

The exclusion criteria were defined as follows: (1) sustaining an injury resulting in absence from training or matches; (2) being transferred or released from the team during the course of the study; (3) failure to complete the required self-report questionnaires on affect, RPE, or sleep on a regular basis; and (4) any diagnosed psychological or neurological condition that could interfere with affective or perceptual responses, as reported in the athlete’s medical records.

A priori sample size estimation was performed using G*Power 3.1 software [21], considering a repeated-measures ANOVA design with a within-between interaction (2 groups × 2 time). The calculation adopted the following parameters: a moderate to large effect size (f = 0.35), alpha error probability (α) set at 0.05, and statistical power (1 − β) of 0.80. The correlation among repeated measures was set at 0.50, and the nonsphericity correction (ε) was assumed to be 1.00. The sample size estimation was guided by previous studies on affective responses in exercise settings, particularly those conducted by Ekkekakis and Lind [22], reporting moderate to large effect sizes. Based on these parameters, the analysis indicated that a minimum of 20 participants would be required to detect a statistically significant interaction effect, with a critical F-value of 4.41, a noncentrality parameter (λ) of 9.80, and actual power of 0.84. This sample size was deemed adequate to support the primary analyses of the study.

### 2.3 Study Design

Data collection occurred daily and was fully integrated into the athletes’ regular schedule, without interfering with the technical or tactical demands imposed by the coaching staff. Each day, prior to the beginning of the training or match session, athletes reported to a designated area where they responded individually to two affective measures. These instruments were administered approximately 30 minutes before the session, using a digital tablet with questionnaires via Google Forms. Following the completion of the session (either training or match), athletes returned for a second round of affective evaluation 30 minutes after the end of the activity, in accordance with validated procedures for minimizing transient fluctuations in exertion perception [23]. Athletes also reported their RPE once per day, always 30 minutes after the session, using the CR10 Borg scale adapted for the session-RPE method [23], and self-reported sleep duration was recorded each morning before physical activity. The club’s performance staff also maintained a centralized training load registry, allowing the daily calculation of weekly load, monotony, and strain for each athlete. All responses were coded, anonymized, and stored in an Excel database supervised by the research team. All data collections were consistently conducted during the morning period, ensuring standardization of circadian influences on affective responses, perceived exertion, and sleep-related variables.

## 3 PROCEDURES

### 3.1 Anthropometric Assessment

Anthropometric measurements were conducted in the first week of the observation period, in the morning. Body mass was measured using a calibrated digital scale (precision: 0.1 kg), and height was measured with a wall-mounted stadiometer (precision: 0.1 cm). All measurements were performed by the same trained evaluator to ensure consistency. Body fat percentage was estimated using the skinfold method, based on the protocol proposed by Pollock and Jackson [24], which is widely validated for male athletes. Skinfold thicknesses were measured at three anatomical sites: triceps, abdominal, and thigh, using a Harpenden caliper (precision: 0.1 mm). The sum of the seven skinfolds was used to estimate body density, which was then converted to body fat percentage using the Siri equation [25]. All measurements were taken on the right side of the body, with each site measured twice and a third measurement taken if the first two differed by more than 1 mm. Table **1** presents the characteristics of the sample.

### 3.2 Affective, Perceived Exertion, and Sleep Responses

The scales were administered, and the data were collected by a single professional from the team's technical committee, who is also one of the authors of this article. The data were organized into spreadsheets containing information about the date, time, and athletes identified by numbers, along with their responses to the scales. Affective responses were measured by FS and FAS before and approximately 30 minutes after training sessions and matches, as well as for the RPE scale.

Affective valence: FS is an 11-point bipolar scale, ranging from “+5” to “-5”, with an anchor of “0” used to measure the affective valence of individuals, in which “+5” represents “very good” and “-5” means “very bad”. In the present study, the athletes were presented with a questionnaire on the Google Forms platform involving the following question: “Answer how you feel at this very moment about your psychological state” [10, 15].

Activation: FAS is a 6-point scale ranging from 1 (low activation) to 6 (high activation). It was collected before and 30 minutes after each training and/or match with the following question: “How aroused do you feel at this moment?” [26].

The athletes answered the following question: “How was the intensity of today's training?” 30 minutes after training and/or matches, using the assessment of the subjective perception of effort that was made by session RPE. It consisted of a table of numbers (0-10) with the corresponding adjectives (“rest” to “maximal”) for the representation of the effort in the exercise [23].

Self-reported sleep duration was assessed daily in the morning, prior to any physical activity. Athletes were individually asked to respond to the question: “How many hours did you sleep last night?”, using a digital form on a tablet device. This approach enabled the continuous tracking of sleep habits throughout the 11-week observation period, facilitating integration with other subjective and load-related variables.

### 3.3 Calculation of Monotony and Strain

To evaluate weekly training load patterns, two derived indices were calculated: monotony and strain [6, 27]. These metrics are based on the session-RPE method [23], which multiplies the athlete's RPE by the session duration in minutes, resulting in a daily training load score (arbitrary units, AU). Monotony was calculated as the ratio between the mean of the daily training loads over the week and the standard deviation of these same daily values: monotony = mean weekly load ÷ standard deviation of mean weekly load. A high monotony score reflects a lack of variability in daily training loads, which may increase the risk of overtraining and injury.

Strain was calculated by multiplying the total weekly training load by the corresponding monotony score: strain = total weekly load × monotony. These calculations were performed for each athlete on a weekly basis, allowing for individualized monitoring of training consistency and cumulative stress throughout the observation period.

### 3.4 Statistical Analysis

Data distribution was assessed using the Shapiro–Wilk test for each variable, stratified by group and time point. Descriptive statistics are presented as mean ± standard deviation (SD), along with coefficient of variation (CV%) and 95% confidence interval (95%CI). To compare the effect of time (pre vs. post) and group (training vs. match), a two-way repeated measures ANOVA was employed, including within-between interaction effects, and a post-hoc Tukey test was used to verify and locate statistical differences for FS and FAS. In addition, one-way ANOVA was used for weekly load, monotony, strain, and hours of sleep. Effect sizes were calculated using partial eta squared (η^2^p) and interpreted as small (≥ 0.01), moderate (≥ 0.06), or large (≥ 0.14), according to Cohen’s guidelines. The Mann-Whitney test was used to compare training loads vs. match loads. Pearson’s correlation coefficients (r) were used to explore relationships among affective responses, RPE, sleep, monotony, and strain across the observation period. All statistical analyses were performed using IBM SPSS Statistics (version 26.0; IBM Corp., Armonk, NY, USA), and significance was set at *p* < 0.05. All figures were created using GraphPad Prism 8.0.

## 4 RESULTS

### 4.1 General Information

The distribution of all dependent variables was examined using the Shapiro–Wilk test, applied separately for each group and time point. The results indicated that most variables exhibited normal distribution, supporting the use of parametric procedures. No severe violations of normality were observed in affective responses, RPE, or self-reported sleep duration. Similarly, the derived training indices (monotony and strain) showed acceptable distributional properties. It is worth noting that the overall dataset of workload values from both matches and training sessions did not follow a normal distribution; therefore, non-parametric analyses were employed.

### 4.2 General Analysis

Significant results across the weeks were obtained through one-way ANOVA for the load (p < 0.0001), monotony (p < 0.0001), strain (p < 0.0001), and hours of sleep (p < 0.0001), as shown in Fig. (**1**). It was observed that until the 8th week, the load oscillated in an undulating way, and after this period, there was a drop until reaching an increase in the final week, with the main difference in the comparison between the 2nd week and the 10th week (p<0.0001). In relation to monotony, a continuous reduction was found up to the 5th week (p<0.0001) and an increase in the 7th week (p<0.0001) until it fell again from the 9th week (p<0.0001). The strain, on the other hand, showed high values until the 3rd week and a wave behavior from the 4th week (p<0.0001). Sleep time suffered small oscillations during the weeks, but from the 7th week, there was a considerable increase compared to weeks 4 (p=0.0427), 10 (p=0.0001) and 11 (p<0.0001). The weekly responses of training load, monotony, strain, and sleep duration showed fluctuations throughout the 11-week period, indicating that internal load and recovery demands varied across the competitive cycle.

**Fig. (2):**
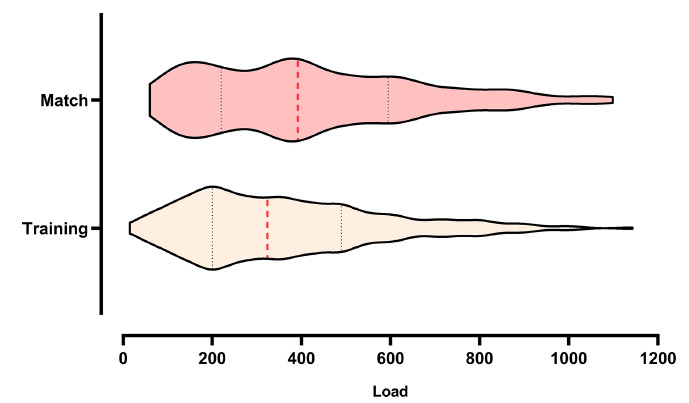
Distribution of internal training load during training sessions and matches. Each dot represents an individual session's load, calculated using the session-RPE method. Red bars indicate the mean and standard deviation for each condition. A wider dispersion and higher extreme values can be observed in match sessions, while training loads show a more concentrated distribution around moderate values.

The Mann-Whitney test showed a significant difference in the match x training comparison (Fig. **2**) for the variables load (p=0.0333) and sleep (p<0.0001), while for the affective variables, no significant values were found (p > 0.05). These findings suggested that matches are perceived as more demanding and have a greater impact on post-session load and sleep duration when compared to training sessions. However, the absence of significant differences in affective responses may indicate a similar affective experience across both types of activity, or reflect individual variability not captured at the group level.

### 4.3 Primary Outcome

**Fig. (3):**
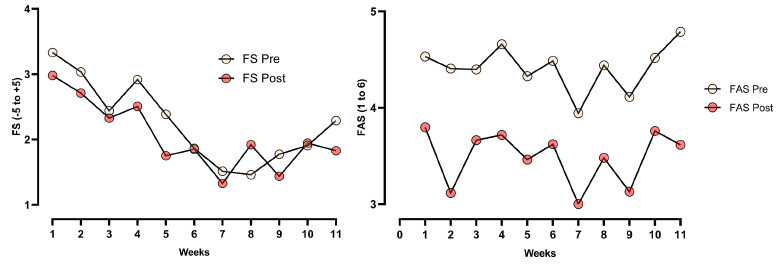
Weekly variation in affective responses measured by the FS (left panel) and the FAS (right panel) before and after training sessions and matches. Affective valence showed a progressive decrease across the weeks, with consistently lower values observed post-session. Arousal levels remained relatively stable pre-session, but they showed a consistent reduction post-session throughout the observation period.

From the data collected during the study weeks, no interaction was observed between week and moment (pre and post) in two-way ANOVA for the FS (p = 0.130; η^2^ = 0.030) or the FAS (p = 0.405; η^2^ = 0.21). However, significant main effects were identified for weeks (FS: p < 0.0001; η^2^ = 0.079; FAS: p < 0.001; η^2^ = 0.075) and for moments (FS: p < 0.001; η^2^ = 0.205; FAS: p < 0.0001; η^2^ = 0.840). There was a decrease in the responses of affective valence (before and after the sessions) until the 7th week (p<0.002), and after that, an increase began to be characterized, while the athletes behaved in a very oscillatory manner, in which the 7th week showed a significant difference with the 3rd week (p<0.003), 4th week (p<0.001), and 11th week (p<0.001). Regarding the behavior of affective variables before and after the sessions, a considerable drop in affective valence (p<0.0001) and in activation (p<0.0001) was observed after the sessions. Figure **3** presents the outcomes of the FS and FAS scales. These results indicated that both affective valence and activation varied across the competitive cycle, with affective responses showing a more consistent temporal trend and activation presenting greater week-to-week variability. The consistent decrease in affective scores after training sessions suggested an acute psychological response to exertion, reinforcing the value of moment-specific affective monitoring.

Individual variability in affective responses showed that affective valence decreased in 71% of the athletes and increased in 29% when comparing pre- and post-match and training sessions. Of the 15 athletes who showed a reduction in affect, 7 of them had an amplitude between 7 and 10 points on the FS, and 8 had an amplitude of 1 to 5 points on the scale both before and after the sessions. In relation to the 6 athletes who had increased affective response after the sessions, 5 athletes presented amplitude of 1 to 5 points before the stimuli, and only 1 presented greater amplitude, while after the sessions, 3 athletes expressed amplitude up to 5 points, and another 3 expressed between 7 and 9 points. These observations illustrated that affective responses varied considerably between athletes, although a majority showed reductions in valence after sessions, suggesting that, even under similar training and match conditions, individual affective responses can differ in both direction and magnitude.

### 4.4 Secondary Outcome

Spearman's correlation showed a significant correlation between affect and traditional variables with small and trivial effect sizes, as shown in Fig. (**4**). In relation to the correlation of affect with the subjective perception of effort and sleep hours, a small correlation was found both before and after the sessions in the valence and activation scales. In relation to monotony and strain, a small correlation was found for affective valence before and after the sessions, while for activation, the correlation was trivial. Although statistically significant, the weak correlations observed suggested that affective responses may reflect aspects of training not fully captured by traditional internal load or recovery markers.

In general, affective responses showed trivial but statistically significant positive correlations with PSE and sleep duration. In contrast, monotony and strain exhibited very weak, non-significant correlations in most cases, with coefficients close to zero. An exception was observed in the negative correlation between FAS Pos and PSE (r = –0.25, p < 0.0001, T), suggesting an inverse relationship between perceived exertion and post-session arousal. Overall, the heatmap revealed a low-to-trivial magnitude of association between affective responses and traditional training load indicators, with sleep duration showing the most consistent, albeit modest, associations. These findings reinforced the notion that affective responses may operate somewhat independently from traditional load markers, with sleep duration showing the most consistent, though still modest, associations.

## 5 DISCUSSION

This study aimed to examine the acute and longitudinal effects of training sessions and competitive soccer matches on affective responses (valence and arousal), RPE, and sleep duration in under-20 male soccer athletes. Additionally, we explored the associations between affective responses and internal training load variables in the competitive cycle. The main findings revealed a significant decline in affective valence and arousal following training and match sessions, with 71% of athletes exhibiting post-session reductions. Despite these affective shifts, correlations with internal load metrics were weak to trivial, and only modest associations were observed with perceived exertion and sleep duration. Notably, sleep duration increased progressively over the weeks, while internal load, monotony, and strain decreased toward the end of the observation period. This integrated monitoring approach may offer a novel contribution to the literature by capturing affective dynamics and subjective recovery throughout a competitive season, reinforcing the role of affect-based markers in optimizing training load management and athlete readiness in youth soccer contexts.

### 5.1 Primary Outcome

Affective valence represents a core dimension of affective response and is considered a sensitive marker of psychophysiological status during exercise [10, 15, 28]. The affective valence may be explained by the combined influence of cognitive and interoceptive regulation pathways [29, 30]. This interaction has been highlighted by Ekkekakis [10], who proposed a dual-process model based on interoceptive and cognitive pathways. According to this framework, physiological stimuli originating from the body, such as changes in cardiorespiratory and metabolic states, are processed automatically and subcortically, primarily by structures, like the amygdala and insular cortex. These signals often produce rapid and negative affective responses, particularly at higher exercise intensities. Conversely, cognitive factors, such as self-efficacy, perceived control, and the personal meaning attributed to the effort, can modulate the affective experience through conscious appraisal, primarily involving prefrontal cortical regions. Therefore, depending on the exercise intensity and individual characteristics, affective valence emerges from the dynamic predominance of these two regulatory systems, being more interoceptively driven under high-intensity conditions and more cognitively influenced during low-to-moderate intensities [19, 31]. In the present study, the affect demonstrated significant responses in both pre- and post-exercise moments, suggesting that proximity to the most critical competition on the calendar, coupled with the cumulative effect of training loads, may have led to progressively more negative affective responses [32]. It is possible that the approach to the competition intensified psychophysiological stress [33], which may have contributed to the cognitive regulation of affect through factors, such as anticipation, pressure, and increased performance focus. Simultaneously, the interoceptive pathway, influenced by accumulated physiological strain, may have played a role in modulating these affective responses. This dual regulation highlights the complex interplay between psychological and physiological factors in shaping athletes' experiences throughout a season, in a manner similar to what has been observed in non-athletes engaging in exercise [29].

Although the current literature addressing affective responses in soccer remains scarce, particularly in studies assessing both pre- and post-session measurements, evidence from high-intensity interval training (HIIT) provides useful parallels. Farias-Júnior *et al.* [12] reported a progressive decline in affective valence during HIIT sessions, with more negative responses emerging as the session progressed. Similarly, Oliveira *et al.* [34] observed post-exercise reductions in arousal among men undergoing both continuous and interval training protocols, indicating that high-intensity intermittent stimuli tend to reduce perceived activation [34-36]. These patterns have mirrored our findings, being particularly relevant given the intermittent nature of soccer [3]. Despite being classified as an aerobic team sport, soccer is characterized by frequent bouts of intense, anaerobic actions interspersed with active recovery periods, features typical of interval-based exercise [1]. This physiological profile may partially explain the decrease in affective valence observed after training sessions and matches in our study. Conversely, in protocols involving continuous moderate-intensity exercise, affective responses appear to follow a distinct trajectory. For instance, Van Landuyt *et al.* [37] and Ekkekakis and Petruzzello [11] documented increases in valence and either stable or reduced arousal 20 minutes post-session, suggesting exercise modality to play a pivotal role in shaping affective trajectories. Taken together, these comparisons reinforced the importance of considering the temporal and physiological structure of the sport when interpreting affective responses.

Beyond the variability induced by the type and structure of the stimulus, affective responses also display substantial interindividual variation. In the present study, while 71% of athletes experienced a decrease in affective valence 30 minutes after training sessions or matches, the remaining 29% exhibited increases, albeit with differing magnitudes of change. These findings underscored the heterogeneous nature of affective regulation, suggesting that identical physical and contextual stimuli can evoke markedly different affective experiences across individuals. This pattern aligned with the theoretical framework proposed by Hall, Ekkekakis, and Petruzzello [38], who emphasized affective responses to exercise to not be universally predictable, but rather shaped by personal, psychological, and contextual factors. Similarly, Van Landuyt *et al.* [37] demonstrated considerable variability in both valence and arousal among participants performing moderate-intensity cycling, challenging the assumption that such exercise universally enhances affective state.

Sleep duration progressively increased from the seventh week onward in the present study, coinciding with a reduction in training load, monotony, and strain. This temporal association suggested that accumulated fatigue and psychological stress from the early phases of the competitive cycle may have impaired sleep initially, with partial recovery occurring as physical demands were tapered closer to competition [39]. Similar findings were reported by Fessi *et al.* [9], who observed professional soccer players to experience lower sleep quality and mood disturbances during periods of high training load, which improved as the season progressed. Malone *et al.* [18] found that players reported longer and more restorative sleep during periods of lower training intensity, reinforcing the influence of internal load on sleep behavior. In our study, the longer sleep duration reported prior to matches compared to training days may have reflected anticipatory rest-seeking behavior or adjustments made by athletes and staff to optimize recovery before competition. These results have highlighted the dynamic and load-sensitive nature of sleep in young athletes and supported its inclusion as a key variable in multidimensional monitoring frameworks.

### 5.2 Secondary Outcome

In our study, affective responses exhibited only weak correlations with other subjective and load-related variables, including perceived exertion, monotony, strain, and sleep duration. An inverse trend but weak correlation was observed between weekly training load and affective valence, indicating that as affective responses became more positive, training loads tended to decrease. Fessi *et al.* [9] found a similar pattern, reporting lower affective responses with higher training loads. Expecting strong associations between affective responses and singular metrics, such as RPE or sleep, may oversimplify the phenomenon. In our study, affective responses exhibited only weak correlations with other subjective and load-related variables, which may reflect the inherently multifactorial nature of affect. Rather than signaling methodological issues, these outcomes have been found to be consistent with theoretical frameworks suggesting affective responses to be modulated by both cognitive appraisals and interoceptive cues, which are not always tightly coupled with training load. As highlighted by Ekkekakis [10], affective experiences in exercise are shaped by a complex interplay of individual, contextual, and physiological factors.

Kuhlman *et al.* [40] monitored male collegiate soccer players throughout an entire competitive season, investigating the relationships among external load variables, RPE-load, and self-reported muscle soreness. The authors found that, although RPE-load exhibited strong correlations with objective physical effort measures, such as total distance covered and the number of accelerations, muscle soreness showed only weak associations with external load. This finding reinforces the notion that different subjective indicators do not necessarily capture the same physiological or emotional dimensions of training.

Throughout the competitive cycle, we also observed a progressive reduction in training load, accompanied by corresponding declines in monotony and strain, two variables intrinsically linked to the consistency and accumulation of training stress [5]. Clemente *et al.* [41] reported substantial variations in weekly training load across a 10-week period, with higher values during the early preseason and a progressive reduction and stabilization during the competitive phase. In this context, training monotony and strain also showed distinct patterns: monotony tended to decrease as the weeks progressed, while strain progressively increased during the initial phase before stabilizing. Interestingly, while RPE remained relatively stable, sleep duration gradually increased over the course of the season, likely reflecting the need to improve recovery as physical demands were adjusted. Moreover, match days were associated with significantly higher internal loads compared to training sessions, while longer sleep durations were reported before matches, possibly indicating strategic recovery practices or behavioral adaptations prior to competition. These patterns highlighted the complex interplay between affective responses and psychophysiological load variables, highlighting the usefulness of multidimensional monitoring approaches in youth soccer, as proposed by Gabbett *et al.* [42]. The athlete monitoring cycle is a practical model designed to guide the interpretation and application of training monitoring data. The cycle consists of four key steps: (1) the external load performed by the athlete, (2) the internal response to that load, (3) the athlete’s perceived well-being, and (4) the athlete’s physical and mental readiness for subsequent training or competition [42]. Each stage informs the next, enabling practitioners to make evidence-based decisions regarding load adjustments, recovery strategies, or additional interventions. By integrating both objective and subjective data, the model supports a more individualized and adaptive approach to training prescription and performance management.

In this context, although this study employed traditional statistical methods to analyze relationships among affective responses, sleep, and training load, future research may benefit from the application of machine learning techniques to improve predictive accuracy. Recent studies have demonstrated the effectiveness of multilayer perceptron models in health-related contexts, such as stroke detection and early prediction of self-care difficulties in children with disabilities [43]. These methods could be valuable for identifying athletes at risk of negative responses, such as reduced affective valence or impaired sleep, before such outcomes manifest. Incorporating predictive modeling into athlete monitoring protocols may enhance the ability to implement timely and individualized interventions.

### 5.3 Limitations

This study has involved several limitations that should be considered when interpreting the findings. One important limitation pertained to the use of self-reported sleep duration, which, although commonly employed in team sport contexts [44], is susceptible to recall bias and social desirability effects. The absence of objective sleep assessment tools, such as actigraphy or polysomnography, considered the gold standard [44], limited the accuracy of the sleep data and its association with training load. Similarly, the exclusive use of subjective measures for affective and exertion responses (*e.g.*, FS, FAS, and session-RPE) restricted the ability to capture the physiological foundations of training adaptations. The lack of objective monitoring tools, such as GPS tracking, heart rate variability, or neuromuscular assessments, was due to logistical and institutional constraints during the observation period. Moreover, external training load parameters (*e.g.*, distance covered, sprint count, and number of accelerations) were not recorded, limiting the integration between objective and subjective indicators of training stress. In addition, external variables known to influence recovery and sleep, such as screen exposure, pre-sleep nutrition, and environmental conditions, were not systematically controlled, and sleep quality was not assessed, which may have reduced the interpretive value of the recovery data. Another methodological limitation was the fixed 30-minute post-session window used to assess affective responses. While based on prior validation studies, this approach may not have captured individual differences in the time course of affective recovery, as some athletes may have exhibited delayed responses. Future studies should consider multiple post-session assessments to better characterize these dynamics. The study’s observational design also precluded causal inferences. Furthermore, the sample was restricted to 21 players from a single under-20 elite soccer team, limiting the generalizability of the findings to other teams, age groups, or competitive levels. Finally, although the 11-week duration reflected the full cycle of preparation and participation in the Copa São Paulo de Futebol Júnior, a key tournament in Brazilian youth football, it may not have represented broader seasonal dynamics. A longer follow-up could improve ecological validity, but roster changes after the tournament may likely compromise the consistency of longitudinal data collection. Despite these limitations, the study has provided contextually relevant insights into psychophysiological responses during a real-world competitive cycle in youth soccer.

### 5.4 Practical Applications

Coaches and support staff may consider using affective assessments before and after training or matches as an additional tool to monitor athletes' psychophysiological responses throughout the season. Although affective responses alone are not sufficient to guide training decisions, they can provide complementary information to support individualized adjustments in load management and recovery strategies. These applications should be considered with caution, given the exploratory nature of the current findings.

## CONCLUSION

There was a significant reduction in affective valence over the weeks. Fluctuations in affective responses were observed across weeks in line with accumulated load and competition demands. Seventy-one percent of athletes showed a decrease in affective valence post-activity. The monotony and strain showed a declining trend over the competitive cycle, particularly in the latter weeks, indicating a reduction in training variability and cumulative load. These reductions coincided with an increase in self-reported sleep duration, suggesting a favorable shift in the balance between training stress and recovery. Affective measures displayed weak correlations with perceived exertion, monotony, strain, and sleep duration.

In summary, while our findings should be interpreted with caution, particularly given the limited scope and observational design of the study, they suggest that tracking affective responses, when integrated with other indicators, may offer additional context to guide individualized training and recovery strategies. Rather than proposing affect as a standalone metric, we advocate for its cautious use as a complementary element in the complex process of managing training load, especially during demanding phases of the season. Finally, future studies should combine subjective and objective measures, include larger and more diverse samples, and better control for contextual variables to improve an understanding of the relationship among training, recovery, and affective responses in athletes.

## Figures and Tables

**Fig. (1):**
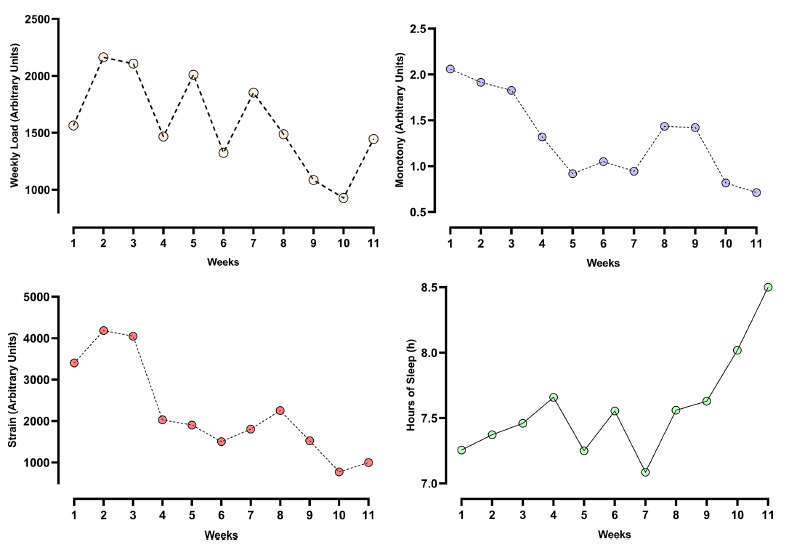
Behavior of the variables weekly load, monotony, strain, hours of sleep, affective valence, and activation. Top left: Weekly training load (session-RPE × duration) fluctuated across the cycle, with a progressive decline toward the final weeks. Top right: Self-reported sleep duration gradually increased, especially from week 7 onward. Bottom left: Monotony (mean daily load ÷ standard deviation) showed a consistent downward trend over time. Bottom right: Strain (weekly load × monotony) followed a similar decreasing pattern, with pronounced reductions during the final phase of the cycle.

**Fig. (4):**
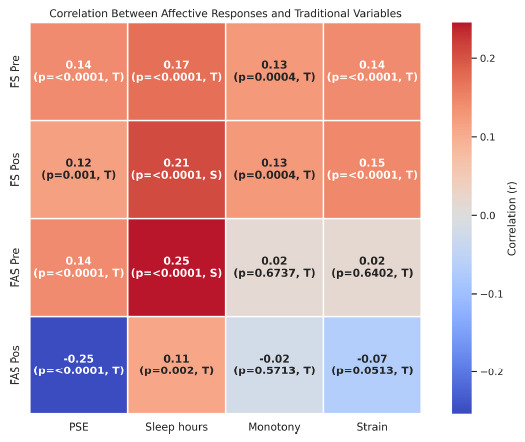
Heatmap showing the correlation coefficients (r) between affective responses, FS and FAS, measured before (Pre) and after (Pos) training sessions and matches, and traditional internal load variables: RPE, self-reported sleep duration, monotony, and strain. Each cell displays the correlation coefficient, significance level (p), and effect size classification, being trivial (T) or small (S).

**Table 1:** Descriptive data of 21 athletes.

	**Mean**	**SD**	**CV**	**95% CI**
Age	18.5	0.64	3%	18.21 – 18.79
Body mass (Kg)	72.92	5.47	7%	70.43 – 75.41
Height (cm)	179.0	7.40	4%	164.6 – 182.3
Body fat (%)	5.40	2.00	37%	4.49 – 6.31

SD = Standard deviation; CV = Coefficient of variation; 95% confidence interval.

## Data Availability

The datasets generated and analyzed during the current study will be available from the corresponding author upon reasonable request.

## LIST OF ABBREVIATIONS

FS: Feeling Scale
FAS: Felt Arousal Scale
RPE: Rating of perceived exertion
AU: Arbitrary units
SD: Standard deviation
CV: Coefficient of variation
ANOVA: Analysis of variance
η^2^p: Partial eta squared

## STANDARDS OF REPORTING

STROBE guidelines were followed.
